# Quadratic programming by spiking neuronal networks

**DOI:** 10.1186/1471-2202-15-S1-P51

**Published:** 2014-07-21

**Authors:** Rubén Moreno-Bote, Philipp Schustek

**Affiliations:** 1 Research Unit, Parc Sanitari Sant Joan de Deu and Universitat de Barcelona, Esplugues de Llobregat, Barcelona, Spain, 08950; 2 Centro de Investigación Biomédica en Red de Salud Mental (CIBERSAM), Esplugues de Llobregat, Barcelona, Spain, 08950

## Introduction

What type of computations can neuronal networks perform? One could reasonable argue that neuronal networks should be able to solve “all” computational problems of interest, as our brain is made of those networks. However, finding specific non-trivial and interesting examples of neuronal networks that solve a particular computational problem has been quite difficult, remarkably. There are very successful examples, though, such a Hopfield networks for memory storage and retrieval, but in particular the computational capabilities of spiking networks have been far less investigated. Finding new examples is important for a better understanding of the functioning of the brain.

## Results

We find that a family of quadratic programming (QP) problems with linear constraints can be solved exactly by networks of integrate-and-fire networks, with the only approximation being finite number of spikes. We show that a network of integrate-and-fire neurons can encode an input vector by approximating it as a linear combination of stored feature vectors weighted by non-negative coefficients. Therefore, the network is able to solve a QP problem with non-negativity constraint on the coefficients. While in previous rate-based implementation of QP problems impose the non-negativity of the firing rate artificially in the dynamical system [1], a neuronal network of interacting neurons satisfies the non-negativity constraint for free. We show that a L1 and L2 priors of the coefficients of the input vectors are encoded in the activity of the network as a constant negative current and a higher hyperpolarizing reset, respectively. We also show that these networks in the presence of probabilistic synapses sample the space of solutions of the QP problem, and that this sampling obeys contrast-invariant properties. Finally, we find that when the feature vectors are dense, the dynamics of the networks have very slow modes, which generate slow transients and as a result large spiking variability. Despite this large variability, the representation of the stimulus is very stable over time. Even in the presence of dense features, L1 regularization reduces spiking variability and allows a very fast convergence to the best solution. When features are not dense, convergence to the best solution is fast regardless of the regularization.

## Conclusions

We have designed a spiking network that can solve exactly a QP problem with linear constraints. The networks are able to decompose an input vector into a linear combination of stored features with non-negative coefficients. This type of networks can be important for applications such as odor identification and classification, and biologically-inspired memory storage.
